# COVID-19 Detection in Chest X-ray Images Using a New Channel Boosted CNN

**DOI:** 10.3390/diagnostics12020267

**Published:** 2022-01-21

**Authors:** Saddam Hussain Khan, Anabia Sohail, Asifullah Khan, Yeon-Soo Lee

**Affiliations:** 1 Pattern Recognition Laboratory, Department of Computer & Information Sciences, Pakistan Institute of Engineering & Applied Sciences, Nilore, Islamabad 45650, Pakistan; hengrshkhan822@gmail.com (S.H.K.); anabia.sohail@gmail.com (A.S.); 2 PIEAS Artificial Intelligence Center (PAIC), Pakistan Institute of Engineering & Applied Sciences, Nilore, Islamabad 45650, Pakistan; 3 Center for Mathematical Sciences, Pakistan Institute of Engineering & Applied Sciences, Nilore, Islamabad 45650, Pakistan; 4 Department of Biomedical Engineering, College of Medical Sciences, Catholic University of Daegu, Daegu 42472, Korea

**Keywords:** coronavirus, COVID-19, SARS-CoV-2, pandemic, X-ray, channel boosting, split-transform-merge, deep learning, CNN, transfer learning

## Abstract

COVID-19 is a respiratory illness that has affected a large population worldwide and continues to have devastating consequences. It is imperative to detect COVID-19 at the earliest opportunity to limit the span of infection. In this work, we developed a new CNN architecture STM-RENet to interpret the radiographic patterns from X-ray images. The proposed STM-RENet is a block-based CNN that employs the idea of split–transform–merge in a new way. In this regard, we have proposed a new convolutional block STM that implements the region and edge-based operations separately, as well as jointly. The systematic use of region and edge implementations in combination with convolutional operations helps in exploring region homogeneity, intensity inhomogeneity, and boundary-defining features. The learning capacity of STM-RENet is further enhanced by developing a new CB-STM-RENet that exploits channel boosting and learns textural variations to effectively screen the X-ray images of COVID-19 infection. The idea of channel boosting is exploited by generating auxiliary channels from the two additional CNNs using Transfer Learning, which are then concatenated to the original channels of the proposed STM-RENet. A significant performance improvement is shown by the proposed CB-STM-RENet in comparison to the standard CNNs on three datasets, especially on the stringent CoV-NonCoV-15k dataset. The good detection rate (97%), accuracy (96.53%), and reasonable F-score (95%) of the proposed technique suggest that it can be adapted to detect COVID-19 infected patients.

## 1. Introduction

COVID-19 is a severe and continuing pandemic, which broke out in December 2019 and has now affected the whole world [1]. This new pathogenic viral infection is caused by a new virus from the coronavirus (CoV) family named SARS-CoV-2. COVID19 is highly transmissible from one individual to another, even before the onset of clinical symptoms [2,3]. COVID-19 causes a respiratory illness that can be asymptomatic, or its clinical manifestation can span fever, cough, myalgia, respiratory impairment, pneumonia, acute respiratory distress and even death in severe cases [4,5]. These factors necessitate the early detection of COVID-19 for proper care of patients and to control infection spread [6]. The standard virus antigen detection approach approved by the World Health Organization is Polymerase Chain Reaction (PCR); however, it suffers from a high False-negative rate depending upon viral load and sampling strategy (30–70% True-positive rate) [7,8,9].

Radiological imaging (X-ray, CT) is used as an assisted screening tool to counter the False-negative rate of PCR in symptomatic patients. It acts as a first-line diagnostic measure for patients suspected of COVID-19 and suffering from a chest infection [10]. In addition to diagnostic importance, X-ray and CT images are used for severity assessment and patients’ follow-up [11]. Chest imaging manifests radiological patterns specific to COVID-19. These patterns commonly include two different types of opacities: ground-glass opacities (GGO) and mixed attenuation (GGO and consolidation). The opacities can be multifocal, patchy, or segmental in distribution, and show multi-lobar and bilateral lung involvement [12]. In patients with severe COVID-19 pneumonia, lung opacity increases, and its characteristic marks become incomprehensible because of consolidation [13]. 

Compared to CT imaging, X-ray imaging is a quick and easy method which is widely available in hospitals at a low cost. Moreover, portable X-ray devices make it easy to perform imaging in deprived areas, field hospitals and intensive care units [14].The visual assessments of radiographic images from COVID-19 patients require trained radiologists. The ongoing pandemic is pacing a considerable burden on a limited number of radiologists. The inevitable importance of a timely diagnosis stresses the need for automated assistance tools that can facilitate radiologists in the initial screening [15].

Deep learning (DL) models are a powerful tool for the analysis of medical images. Several DL models have shown promising performance for detecting COVID-19 [16,17,18]. Various techniques have been developed for automated COVID-19 diagnosis in X-ray images [19,20]. However, most of the researchers exploited existing CNNs designed to classify natural images for COVID-19 detection. Natural images are often represented by large sized well-defined objects, contrary to COVID-19 radiographic patterns, which are normally exhibited by obscure lung markings and patches of opacity and consolidation. The exploration of image content by considering COVID-19 radiographic patterns of region-homogeneity, textual variation, boundaries, etc., can result in enhanced COVID-19 detection tools. Additionally, most of the work exploited a single dataset source with a limited number of images for model training and evaluation. The evaluation of a small dataset is likely to show over-optimized performance.

DL architectures have shown excellent performance in the medical and commercial fields [21,22,23,24,25]. Therefore, DL is primarily employed in the detection of COVID-19 infection and drug repurposing in diverse ways [26,27,28,29]. Several researchers have employed CNN to speed up the analysis of COVID-19 infected images [30,31,32,33,34]. Initially, COVID-19 labelled datasets were small in size and generally not suitable for practical implementation. Therefore, the existing pre-trained CNN architectures such as VGG, ResNet, Inception, etc., have been employed in the COVID-19 classification challenge. These architectures have been fine-tuned on a problem-specific COVID dataset using TL and achieved optimal results. However, because of the non-availability of the consolidated data repository, these models have been evaluated on various small-sized datasets gathered from GitHub and Open-I respiratory, etc.

In one of the early works, a ResNet-50 model using TL has been fine-tuned on small X-ray chest data and achieved 98% accuracy [30]. Similarly, a ResNet-101 architecture has been used to detect abnormality in X-ray images using various datasets and reported sensitivity of 0.77 and accuracy of 71.9% [35]. Moreover, a pre-trained inception network has been employed to predict COVID-19 and reported accuracy of 89.5%) [36]. These models have been employed on multi-class problems such as Healthy, COVID-19 non-affected patients and COVID-19 affected pneumonia patients. However, the aforementioned techniques have been trained on natural images and fine-tuned on medical datasets, which affects COVID-19 detection performance.

Similarly, 19 layers of deep CNN architecture have been developed based on the idea of ResNet, named COVID-Net and employed on the same dataset [14]. The COVID-Net model showed good accuracy (92%) but at a low detection rate (sensitivity) (87%). Similarly, COVID-CAPS has been designed based on the concept of Capsule Net and achieved accuracy of 98%, sensitivity of 0.80, and AUC of 0.97 [37]. The darknet network has been proposed to diagnose COVID infection accurately on the complex dataset. In this work, darknet performed both binary (COVID-19 vs. Healthy) and multi-class (COVID-19, NonCOVID-19, and Healthy) discrimination. Darknet achieved a detection accuracy of 98% and 87% for binary and multi-class, respectively [38].

In the most recent studies, a framework of four pre-trained existing CNN networks has been utilized to identify the presence of COVID-19 in X-rays. These models (ResNet18, ResNet50, Squeeze Net, and DenseNet121) are fine-tuned on the COVID-Xray-5k dataset. On average, thy obtained a detection rate of approximately 98% [39]. A pre-trained CNN model like ResNet-50 has also been used for deep feature extraction and ML classification (SVM). This model is fine-tuned on a small COVID-19 dataset using TL and reported an accuracy of 95% [40]. Similarly, a pre-trained ResNet-152 has been used for deep feature extraction in combination with Random Forest and XGBoost classifiers, achieving accuracy of 97.3% and 97.7%, respectively [41]. These models have been employed on the imbalanced dataset and reported their accuracy and sensitivity results, which are not considered appropriate for imbalanced data.

In this regard, we developed a new stringent COVID-19 chest X-ray dataset by collecting samples from multiple sources to ensure the generalization of the proposed customized CNN models and evaluated in terms of standard performance metrics. In this work, our aim is to focus on extracting the radiological patterns from X-ray images by developing a new convolutional block based on the idea of split–transform–merge (STM). This new block systematic implements Region- and Edge-based (RE) operations at each transformation branch. The proposed custom CNN is named “STM-RENet”. These radiological patterns may be due to different opacities such as Ground Glass Opacity, Consolidation, and Reticulation [13]. In STM-RENet, the region operation helps learn about the infectious regions’ characteristics by exploring regions with homogeneous properties and highlighting textural and intensity variations. In addition, edge operation demarcates infected areas and identify lung markings and de-markings in both healthy and COVID-19 infected X-ray images. The representational power of the proposed STM-RENet is augmented by developing a new channel boosted CNN “CB-STM-RENet”, where two additional CNNs generate diverse auxiliary channels using transfer learning (TL). The significant contributions of this research are:

1. A novel CNN block based on the Split-Transform-Merge (STM) concept is developed that systematically exploits the idea of region and edge-based feature extraction in each block of the proposed STM-RENet.
2. The systematic utilization of the region and edge-based implementation at each branch of the new STM block is able to capture the diverse set of features at various levels, especially those related to region–homogeneity, textural variations, and boundaries of the infected region.
3. The idea of Channel Boosting exploited using TL generates diverse auxiliary channels and thus enhances the performance of the proposed STM-RENet.
4. Three different datasets from the publicly available chest X-ray images are generated (CoV-Healthy-6k, CoV-NonCoV-10k, and CoV-NonCoV-15k) and the performance of the proposed techniques is validated.

The layout of the manuscript is ordered as follows. Dataset description, detail of the proposed deep CNN techniques for COVID-19 screening, and experimental setup are given in Section 2. Section 3 explains the results and comparative studies. While Section 4 discusses the results. Lastly, Section 5 concludes.

## 2. Material and Method

### 2.1. Dataset

**Figure 1:**
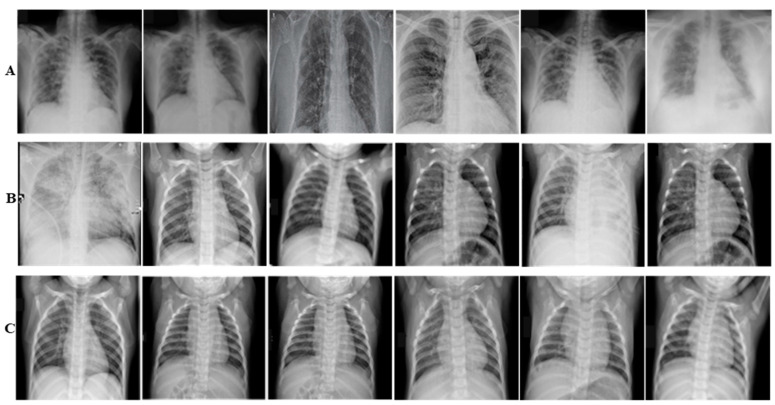
Panel (**A**–**C**) illustrate COVID-19, Non-COVID-19, and Healthy X-ray images, respectively.

COVID-19 is a new challenge, and to the best of our knowledge, up till now no consolidated data has been available. Consequently, we collected radiologists’ authenticated X-ray images from different publically accessible data repositories. The details of the datasets are mentioned in this section. The examples of X-ray images from the assembled dataset are illustrated in Figure 1.

#### 2.1.1. CoV-Healthy-6k Dataset

For this study, initially, a COVID-19 vs. Healthy individuals’ dataset has been built. The COVID-19 X-ray images used in this research are collected from [42]. The Healthy individuals’ dataset is obtained from [39] and the Kaggle repository [43]. The accessed repositories contain radiologists’ verified X-ray images from multiple standard accessible sources and hospitals. The new dataset consisted of 3224 images from both COVID-19 infected and Healthy individuals. The advantage of using an open-source dataset is that it can easily be exploited by other researchers to quickly implement different DL models in order to assess the capability of the proposed CB-STM-RENet technique.

#### 2.1.2. CoV-NonCoV-10k Dataset

This dataset consisted of COVID-19 infected and non-COVID-19 patients’ data. The non-COVID-19 includes both Healthy and other viral infected X-ray images. These X-ray images are collected from [42], whereas the same set of Healthy samples are also used as defined in the CoV-Healthy-6k Dataset. In non-COVID-19 samples, pneumonia and other respiratory infections are caused by different viral and bacterial infections other than COVID-19. This dataset contains a total of 9538 images, out of which both the COVID-19 and non-COVID-19 class includes 4769 images.

#### 2.1.3. CoV-NonCoV-15k Dataset

We have also built a stringent dataset to assess the robustness of the developed technique. For this, the CoV-NonCoV-10k dataset is augmented by including additional samples from [39]. This new CoV NonCoV-15k dataset is imbalanced and consists of 15,127 total images, out of which 5223 and 9904 images are from COVID-19 infected and non-COVID-19 individuals, respectively.

### 2.2. Experimental Setup

#### 2.2.1. Dataset Division

A holdout cross-validation scheme was used for the training and evaluation of the deep CNN models. The dataset was divided into train and test sets at a ratio of 8:2. From the training dataset, 20% was reserved for model validation and hyper-parameter selection. The final evaluation of the model was made on the test set, which is kept separate from the training and validation datasets.

#### 2.2.2. Pre-Processing

DL models usually overfit on a small size dataset. Therefore, sufficient volume of data is essential for efficient learning and for improving model generalization. Data augmentation is a reasonable way of enhancing the generalization of the model by incorporating multiple variations in the base dataset. The training samples in this study were augmented by applying different types of transformations, including horizontal and vertical reflections, rotation, and shear. All the images were resized to 224 × 224 × 3 before assigning to CNN for training.

#### 2.2.3. Model Implementation Details

Deep CNN models were implemented in an end-to-end method. SGD was employed as an optimizer function to reduce cross-entropy loss. Softmax was employed for the identification of class probabilities. The training was managed using a Piecewise learning rate scheduler by setting learning rate value as 0.0001 and momentum as 0.95. Some of the CNN Models were trained with a batch size of 16, while others were trained with a batch size of 32 for 10 epochs. For each of the CNN models, a 95% confidence interval (CI) was computed [44]. The training time for 1 epoch on NVIDIA GeForce GTX Titan X was ~1–2 h. All the implemented models were trained for all three different datasets and evaluated on their unseen test sets.

#### 2.2.4. Working Environment

Deep CNN models were built in MATLAB 2019b, and simulations were performed using the DL library. All the experimentations were done on a CUDA enabled NVIDIA GeForce GTX Titan X computer, with 64 GB RAM.

### 2.3. Deep Channel Boosted STM-RENet for COVID-19 Detection

**Figure 2:**
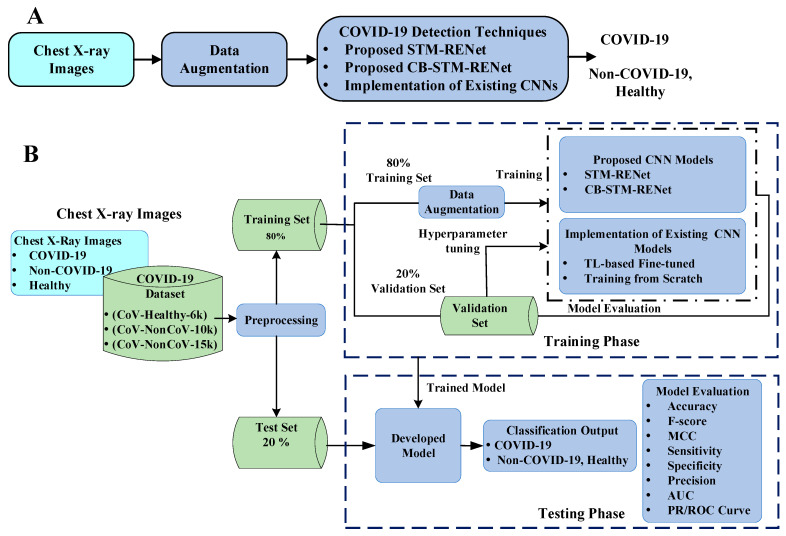
Panel (**A**,**B**) depict the brief and detailed workflow for the proposed COVID-19 detection framework, respectively.

This study proposes two new CNN based techniques for the screening of COVID-19 in X-rays. The proposed techniques target the discrimination of COVID-19 infected from both Non-COVID-19 and Healthy individuals. In this regard, a new CNN classifier based on novel split-transform-merge (STM) block [45] is developed, which systematically implements RE-based operations for the learning of COVID-19 specific patterns and terms, as “STM-RENet”. This architecture is also known as “PIEAS Classification Network-4 (PC Net-4)”. The representation strength of the proposed STM-RENet is enhanced using Channel Boosting to improve the detection rate while maintaining high precision. The proposed Channel Boosted STM-RENet is termed “CB-STM-RENet” or “PIEAS Classification Network-5 (PC Net-5)”. The proposed techniques’ performances are compared with several existing CNNs by implementing them from scratch and adapting using TL. The workflow is illustrated in Figure 2. We have shared code related to this work at https://github.com/PRLAB21/COVID-19-Detection-System-using-Chest-X-ray-Images accessed on 12 December 2021.

#### 2.3.1. Proposed STM-RENet

Deep CNNs have been largely utilized in image processing applications because of their strong pattern mining ability [46,47]. According to the target medical image analysis, CNN exploits the image’s structural information using convolution operation and dynamically extracts feature hierarchies. The exploitation of innovative ideas in CNN design has increased their use in medical image classification, detection and pattern discovery tasks [48].

**Figure 3:**
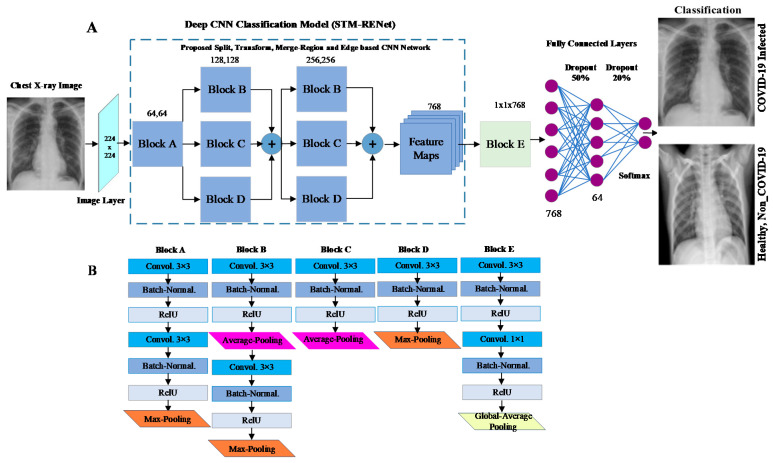
Panel (**A**) illustrates the proposed STM-RENet technique, while (**B**) shows detail of its blocks.

This work proposes a new COVID-19 pneumonia-specific CNN architecture based on the novel split-transform-merge block (STM) and RE-based feature extraction operations [49,50]. RENet systematically implements region and edge-based operations which may capture region homogeneity and boundary features of the COVID-19 infected region at various levels. This new architecture is named STM based RENet (STM-RENet) and illustrated in Figure 3.

The proposed block consists of three sub-branches. The concept of RE-based feature extraction is systematically employed at each branch using average and max-pooling in combination with the convolutional operation to capture discriminating features at a high level. The discriminating features may include region homogeneity, boundaries pattern, and textural variation. The output channels or feature maps of each convolutional operation are 64, 128 and 256, respectively.

In Equation (1), the convolution operation is utilized. Input and the resultant channel are denoted by **x**. M × N and p × q is the dimension of channel and filter, respectively. Equations (2) and (3) demonstrate the average (**x**^avg^) and max-pooling (**x**^max^) operations, where ‘*s*’ represents stride size. The STM-RENet mines the patterns from the X-ray dataset by splitting the input into three branches. It learns the region-specific variations and their characteristic boundaries using a RE-based operator. Finally, it merges the output from multiple paths using a concatenation operation, which may capture textural variation. In STM-RENet, two STM blocks with the same topology are stacked one after another to extract a diverse set of abstract level features. This idea helps the STM-RENet in extracting a diverse set of variations in the input feature maps.

(1) $$x_{m,n}=\overset{p}{\underset{a=1}{\sum }}\overset{q}{\underset{b=1}{\sum }}x_{m+a-1,n+b-1}f_{a,b}$$

(2) $$x^{\mathrm{avg}}{}_{m,n}=\frac{1}{w^{2}\;}\;\overset{s}{\underset{a=1}{\sum }}\overset{s}{\underset{b=1}{\sum }}x_{m+a-1,n+b-1}$$

(3) $$x^{\max}{}_{m,n}=\max_{a=1,\ldots ,s,b=1,\ldots ,s}x_{m+a-1,n+b-1}$$

#### 2.3.2. The Proposed Deep Channel Boosted STM-RENet (CB-STM-RENet)

Radiographic data exhibits large variations in images, and thus a robust CNN model is required for good discrimination. The discrimination ability of the proposed STM-RENet is enhanced by exploiting Channel Boosting. The idea of Channel Boosting helps in solving complex problems by considering multiple data-representations from different sources [51,52]. In the proposed technique, Channel Boosting is performed by generating auxiliary feature channels from two pre-trained networks via TL to improve the performance of STM-RENet.

##### Significance of Using Transfer Learning (TL)

TL is a type of machine learning that allows to leverage of the knowledge of existing techniques for new tasks. TL can be exploited in different ways for multiple tasks, but the most commonly employed approaches for knowledge utilization are (1) instance-based TL, (2) feature-space based TL, (3) parameter exploitation based TL, and (4) relation-knowledge based TL [53].

Feature space-based TL is often used for solving image classification and pattern recognition related tasks. Pre-trained architecture is adapted to the target domain by fine-tuning network layers or adding additional layers according to the target domain task [54]. This is also commonly known as domain adaptation. Supervised domain adaptation-based TL using pre-trained deep CNNs has been substantially adopted for solving medical imaging tasks. This can help provide a useful set of feature descriptors learnt from the source domain to be effectively applied in a target domain by adapting to the target task via fine-tuning. This reduces the calibration efforts (hyper-parameter selection) which are particularly difficult in deep CNNs because of the vast number of hyper-parameters and considerable training time [46].

##### Significance of Using Auxiliary Channels

CNNs with varied architectural designs have different feature learning capacities. Multiple channels learnt from different deep CNNs exhibit multi-level information. These channels represent different patterns, which may help in precisely explaining class-specific characteristics. A combination of diverse-level abstractions learned from multiple channels may improve the image’s global and local representation. The concatenation of original channels with auxiliary channels gives the idea of an intelligent feature–space-based ensemble, whereby the single learner takes the final decision by analyzing multiple image specific patterns [55].

##### Proposed Channel Boosted Architectural Design

**Figure 4:**
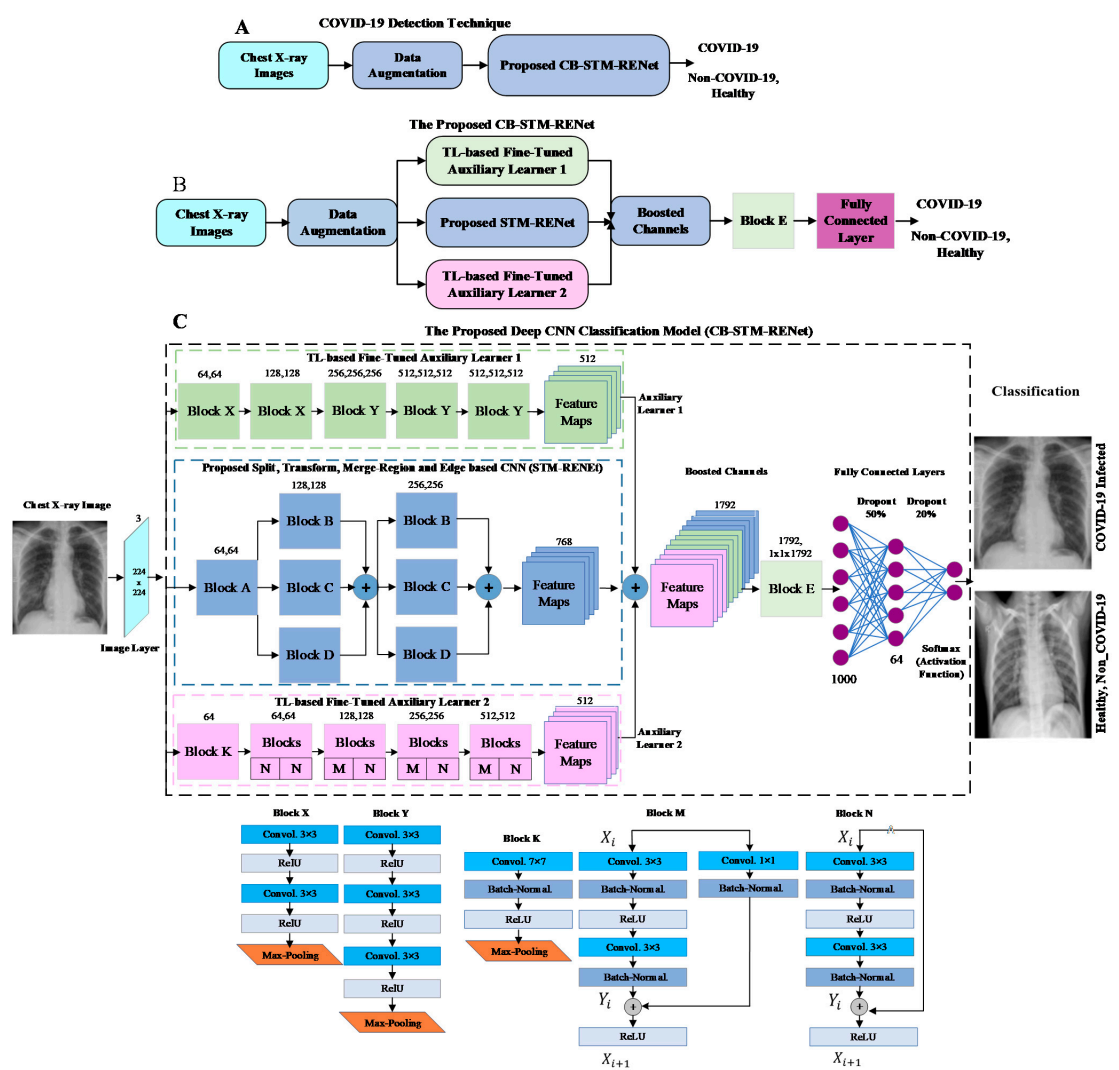
Panel (**A**–**C**) illustrate the brief and detailed proposed CB-STM-RENet COVID-19 detection technique, respectively.

In this work, we utilized supervised domain adaptation-based TL by exploiting two different pre-trained deep CNNs. These deep CNNs vary in architectural design, enabling each model to learn other feature descriptors and encapsulate diverse radiological information from chest X-rays. These two fine-tuned deep CNNs are termed auxiliary learner-1 and -2. The purpose of Channel Boosting is to improve the discriminative capability of the proposed CB-STM-RENet model. The architectural details of CB-STM-RENet are illustrated in Figure 4.

(4) $$C_{\mathrm{Boosted}=}h\left(C_{\mathrm{STM}}||C_{\mathrm{Aux}1}||C_{\mathrm{Aux}2}\right)$$

In Equation (4), **C**_STM_ shows the STM-RENet original channels, whereas **C**_Aux1_ and **C**_Aux2_ are the auxiliary channels generated by TL-based based fine-tuned auxiliary learner 1 and 2, respectively. *h*(.) concatenates the original STM-RENet channels with the auxiliary channels to generate the Channel Boosted input **C**_Boosted_ for the classifier. The boosted channels are provided to convolutional block E, as shown in Figure 3 and Figure 4. At the end of convolutional block E, global-average pooling is employed to minimize the connection intensity. Finally, the fully connected layers are employed to preserve the prominent features, and dropout layers are used to reduce overfitting.

#### 2.3.3. Implementation of the Existing CNNs

For a rigorous assessment of the proposed technique, several existing deep CNNs (Alexnet, VGG-16, VGG-19, Google Net, Inceptionv3, Resnet-18, Resnet-50, Squeeze Net, DenseNet-201, ShuffleNet, Xception) have been implemented [45,56,57,58,59]. These architectures are initially trained from scratch on X-ray data for a fair comparison with the proposed technique. The implemented deep models are computationally intensive and require a sufficient amount of data. Therefore, TL is exploited to optimally train existing CNN techniques and achieve substantial performance on a small amount of data. TL is a type of machine learning in which models already pre-trained for some tasks are used for new tasks by fine-tuning layers of the network or adding some new target-specific layers [60]. In this regard, CNN models trained on ImageNet (natural data) are fine-tuned using TL on X-ray data for binary classification.

## 3. Result

**Figure 5:**
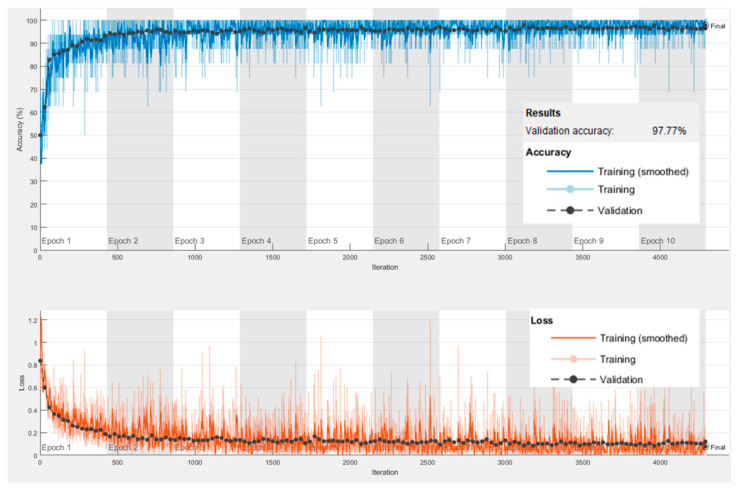
Training and Validation accuracy of the proposed CB-STM-RENet technique on the CoV-NonCoV-15k dataset.

The proposed techniques’ performances are assessed using several performance measures on an unseen test set and benchmarked against well-known existing techniques. Learning plots of the proposed CB-STM-RENet, showing accuracy and loss values for the training and validation set, are shown in Figure 5. The learning plots suggest that the proposed CB-STM-RENet technique quickly converges to optimal values.

### 3.1. Performance Evaluation Metrics

The discrimination ability of the proposed technique is assessed using accuracy and AUC-ROC curve for a balanced dataset. In contrast, the F-score and AUC of precision and recall (PR) curve are used as a performance metric for an imbalanced dataset. Mathew Correlation Coefficient (MCC) is also computed for unbiased estimation as it considers all the examples from COVID-19 positive and negative classes, including both True (TP, TN) and False (FN, FP) predictions. COVID-19 negative class includes both Healthy and non-COVID-19 infected individuals. The details of the qualitative measures, such as sensitivity, specificity, precision, TP, TN, FN, and FP are also reported. COVID-19 positive and negative classes that are truly predicted are known as TP and TN, respectively. Similarly, positive and negative class examples that are misclassified are referred to as FP and FN, respectively. These performance metrics are mathematically expressed in Equations (5)–(10). Accuracy defines the ratio of COVID-19 positive and COVID-19 negative samples that are correctly classified. COVID-19 negative can be Healthy individuals or patients having other viral/bacterial infections. F-score is a measure of accuracy for the imbalanced dataset. Sensitivity and specificity refer to the ratio of COVID-19 positives and negative patients, respectively that are correctly identified. Precision is the proportion of COVID-19 positive predictions made which are actually correct. In Equation (11), the Standard Error (S.E.) at 95% CI is reported for sensitivity because the primary concern is to improve the true positive rate while reducing False-Negative for COVID19 patients’ screening [44]. Here, z = 1.96 for S.E at 95% CI.

(5) $$\mathrm{Acc}=\frac{\mathrm{True}\;\mathrm{COVID}-19\;\mathrm{positives}\;\left(\mathrm{TP}\right)+\mathrm{True}\;\mathrm{COVID}-19\;\mathrm{negatives}\;\left(\mathrm{TN}\right)}{\mathrm{Total}\;\mathrm{Images}\;\left(\mathrm{TP}+\mathrm{TN}+\mathrm{FP}+\mathrm{FN}\right)}\times 100$$

(6) $$\mathrm{Sen}=\frac{\mathrm{True}\;\mathrm{COVID}-19\;\mathrm{positives}\;\left(\mathrm{TP}\right)}{\mathrm{Total}\;\mathrm{COVID}-19\;\mathrm{positive}\;\mathrm{Images}\;\left(\mathrm{TP}+\mathrm{FN}\right)}\times 100$$

(7) $$\mathrm{Spe}=\frac{\mathrm{True}\;\mathrm{COVID}-19\;\mathrm{negatives}\;\left(\mathrm{TN}\right)}{\mathrm{Total}\;\mathrm{COVID}-19\;\mathrm{negative}\;\mathrm{Images}\;\left(\mathrm{TN}+\mathrm{FP}\right)}\times 100$$

(8) $$\mathrm{Pre}=\frac{\mathrm{True}\;\mathrm{COVID}-19\;\mathrm{positives}\;\left(\mathrm{TP}\right)}{\mathrm{True}\;\mathrm{COVID}-19\;\mathrm{positives}\;\left(\mathrm{TP}\right)+\mathrm{False}\;\mathrm{COVID}-19\;\mathrm{positives}\;\left(\mathrm{FP}\right)}\times 100$$

(9) $$F-\mathrm{Score}=2\times \frac{\mathrm{Pre}\times \mathrm{Sen}}{\mathrm{Pre}+\mathrm{Sen}}$$

(10) $$\mathrm{MCC}=\frac{\left(\mathrm{TN}\times \mathrm{TP}\right)-\left(\mathrm{FN}\times \mathrm{FP}\right)}{\sqrt{\left(\left(\mathrm{FN}+\mathrm{FP}\right)\left(\mathrm{FP}+\mathrm{TP}\right)\left(\mathrm{FN}+\mathrm{TN}\right)\left(\mathrm{FP}+\mathrm{TN}\right)\right)}}$$

(11) $$\mathrm{CI}=z\sqrt{\frac{\mathrm{error}\left(1-\mathrm{error}\right)}{\mathrm{Total}\;\mathrm{Samples}}}$$

### 3.2. Performance Analysis of CoV-Healthy-6k

Classification results of the proposed STM-RENet with and without Channel Boosting on the test set of CoV-Healthy-6k are illustrated in Table 1. The discrimination ability in terms of accuracy (STM-RENet: 97.98%, CB-STM-RENet: 98.53%), F-score (STM-RENet: 0.98, CB-STM-RENet: 0.98) and MCC (STM-RENet: 0.96, CB-STM-RENet: 0.97) show that both models can effectively differentiate COVID-19 infected from Healthy individuals. Finally, we perform an ablation study on CoV-Healthy-6k to explore the effectiveness of each auxiliary learner in the proposed STM-RENet. The combined impact of these auxiliary channels on the performance of the proposed technique is also investigated. The empirical results are summarized in Table 2.

### 3.3. Performance Analysis on CoV-NonCoV-10k

The proposed technique is accessed for its effectiveness in discriminating COVID-19 from Non-COVID-19 infected. Therefore, STM-RENet with and without Channel Boosting is trained on CoV-NonCoV-10k with the same set of parameters and evaluated on the test dataset. Table 1 illustrates the classification results. The performance analysis using various evaluation metrics (accuracy: 97.48%, F-score: 0.98, and MCC: 0.95) clearly demonstrates that Channel Boosting significantly improves the discrimination ability of CNN (shown in Table 1).

### 3.4. Performance Analysis on the Stringent CoV-NonCoV-15k

**Figure 6:**
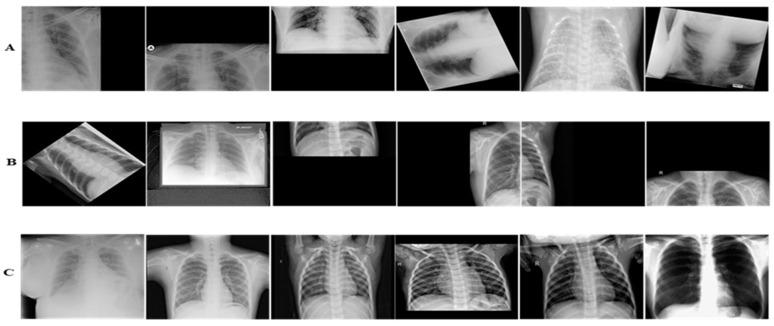
Panel (**A**–**C**) illustrate COVID-19, Non-COVID-19, and Healthy Chest X-ray images, respectively. The images are tough to classify because of having high illumination, translational, rotational, occlusion and missing informational effects.

The proposed technique’s generalization is accessed by evaluating the performance on the stringent CoV-NonCoV-15k dataset, as shown in Figure 6. This dataset is imbalanced and contains a smaller number of COVD-19 positive patients than non-COVID-19 and Healthy individuals, both in training and test sets. Table 1 shows the detection results. F-score and AUC show good learning potential and strong discrimination ability of our proposed CB-STM-RENet technique compared to STM-RENet.

### 3.5. Comparative Analysis with the Existing CNNs

The significance of the proposed architecture and Channel Boosting is explored by implementing existing well known deep CNN techniques. In this regard, different CNN architectural designs, including ShuffleNet, Inception, AlexNet, DenseNet, Xception, VGG, and ResNet, are implemented from scratch and fine-tuned using TL. The results for the best performing existing techniques on a test set of CoV-Healthy-6k, CoV-NonCoV-10k and CoV-NonCoV-15k are shown, in Table 3. In contrast, detailed results of all the implemented techniques on CoV- Healthy-6k are depicted in Table 4. The evaluation metrics suggest that TL improves the learning of discriminating patterns for COVID-19 classification.

Well known CNN architectures such as ResNet, DenseNet, and SqueezeNet have been implemented by Minaee et al. on the COVID-Xray-5k dataset [36]. The dataset (CoV-NonCoV-15K) developed in this study also includes the COVID-Xray-5k dataset and the different datasets mentioned in Section 3.3. Table 5 shows the performance of these models on the CoV-NonCoV-15K dataset. The results show that SqueezeNet gives a low detection rate.

## 4. Discussion

**Figure 7:**
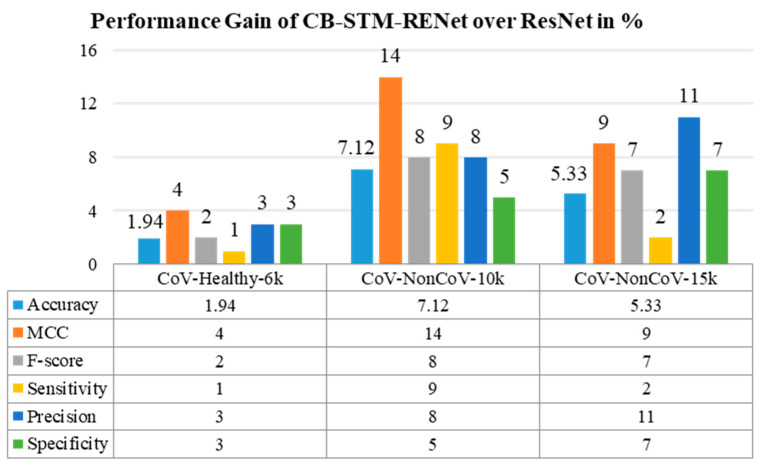
Performance gain of the proposed CB-STM-RENet COVID-19 detection techniques as compared with the ResNet.

**Figure 8:**
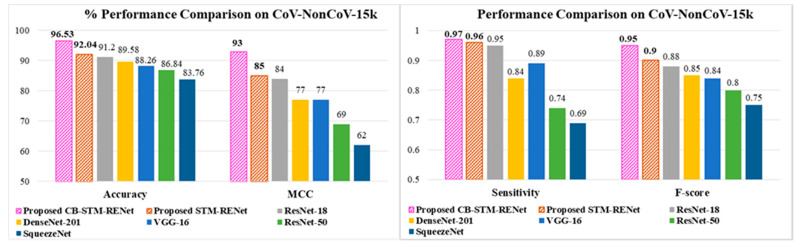
Performance comparison of the proposed CB-STM-RENet and STM-RENet with the well-established models.

The performance comparison based on accuracy, F-score and MCC suggest that the proposed STM-RENet with and without Channel Boosting outperformed the existing techniques, as shown in Table 1, Table 2, Table 3, Table 4 and Table 5. The performance gain of the proposed CB-STM-RENet when compared to the highest performing existing CNN technique (ResNet) is illustrated in Figure 7. The proposed CB-STM-RENet shows a significant improvement in performance as compared to standard CNNs in terms of MCC (13–35%), sensitivity (4–30%), precision (12–18%), F1-Score (10–23%), accuracy (7–15%), specificity (4–10%), PR-AUC (4–14%), and ROC-AUC (2–7%), as depicted in Figure 8. These results demonstrate that the proposed CB-STM-RENet effectively learns region homogeneity, boundaries, and textural variation, which improve COVID-19 classification performance.

### 4.1. Feature Space Visualization

**Figure 9:**
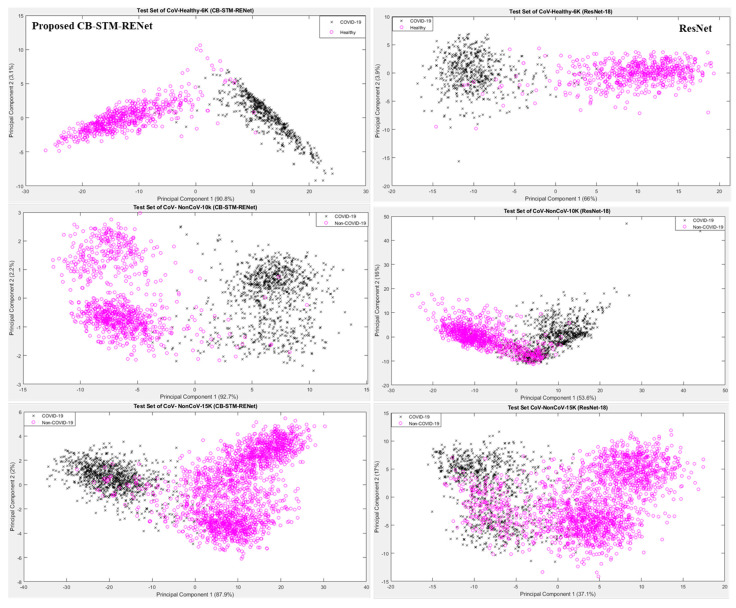
Feature space visualization of the proposed CB-STM-RENet and ResNet on test dataset.

**Figure 10:**
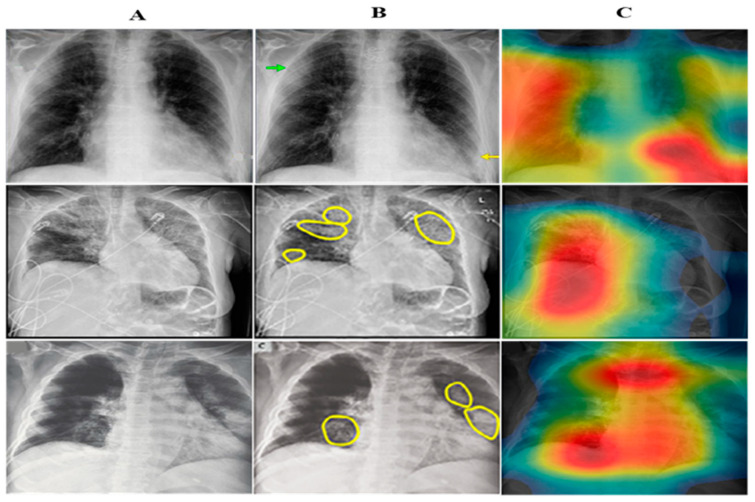
Panel (**A**) illustrates X-ray images. In panel (**B**), COVID-19 infected region is highlighted with an arrow or yellow circle by radiologists. Panel (**C**) shows the heat map of the proposed CB-STM-RENet.

Feature space learnt by the proposed CB-STM-RENet technique is explored for the radiologist’s better understanding and easy decision-making. Figure 9 shows the projection of the first two principal components (PC) of feature space learned by CB-STM-RENet and ResNet for the test dataset. It is evident from 2D plots that the proposed CB-STM-RENet shows the highest discriminative capability (segregation of COVID-19 positive and Non-COVID-19) compared with ResNet on the test datasets CoV-Healthy-6k, CoV-NonCoV-10k and CoV-NonCoV-15k, respectively, while Figure 10 shows the heat map generated by the proposed CB-STM-RENet for the COVID-19 infected region.

### 4.2. Detection Rate Analysis

**Figure 11:**
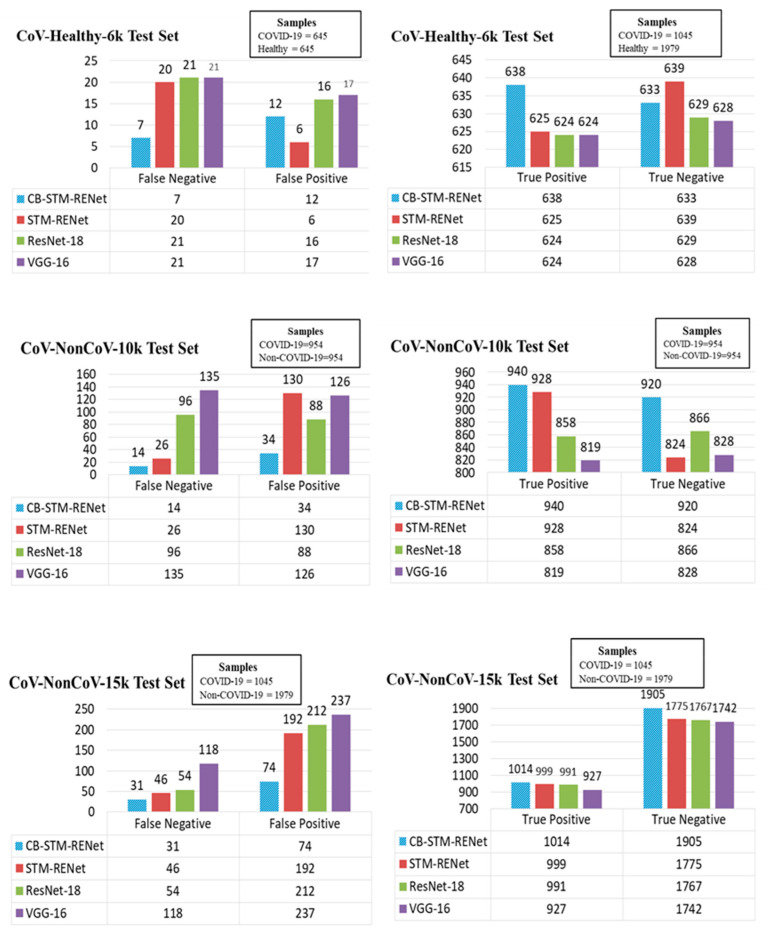
Detection and misclassification rate analysis of the proposed CB-STM-RENet with the existing CNN models.

A significant detection rate is needed for COVID-19 screening, thus limiting infection spread and patient treatment. Therefore, the detection rate (number of correctly identified COVID-19 positive patients) is explored along with the misclassification rate for the proposed technique on all three test sets. The detection and misclassification rate for the proposed CB-STM-RENet and best performing existing techniques are reported in Figure 11, whereas Table 1 and Table 3 show the sensitivity and precision. The quantitative statistics exhibit that the proposed technique with and without Channel Boosting achieved the highest detection rate (ranging from 96–99%) with the minimum number of False positives.

Additionally, CB-STM-RENet significantly decreases the number of False negatives and positives, as shown in Figure 11. The significant precision (ranging from 93–98%) suggests that our proposed technique with Channel Boosting significantly reduced the miss-detection rate (ranging from 1–7%) and can screen the individuals precisely (Table 1, Figure 11). High precision means very few Healthy individuals or non-COVID-19 patients will be Falsely diagnosed with COVID-19 infection, resulting in a lessening of the burden on radiologists.

### 4.3. Evaluation of Diagnostic Ability of the Proposed Technique

**Figure 12:**
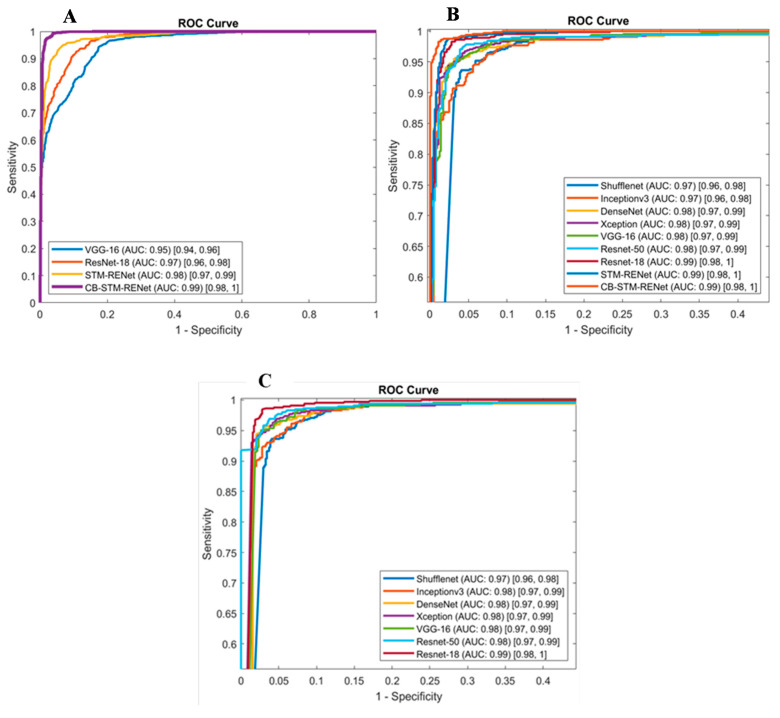
ROC Curve for the proposed STM-RENet, CB-STM-RENet, and existing CNN techniques. (**A**) is reported for CoV-NonCOV-10k, while (**B**,**C**) for CoV-Healthy-6k. ROC in (**C**) is reported for TL-based CNN architecture. AUC CI at 95% is shown in square brackets.

**Figure 13:**
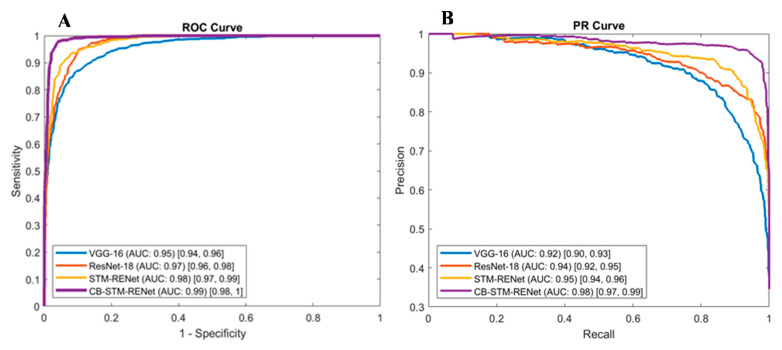
ROC and PR Curve for the proposed CB-STM-RENet, STM-RENet, and existing CNN techniques are reported in panel (**A**,**B**) for CoV-NonCOV-15k. AUC CI at 95% is shown in square brackets.

ROC and PR curves have a significant role in accessing the appropriate diagnostic cutoff for the classification. These curves graphically illustrate the discrimination ability of the classifier at a whole range of possible values [61]. Figure 12 shows ROC curves for the proposed and existing techniques for CoV-Healthy-6k and CoV-NonCoV-10k datasets, whereas the PR curve, in addition to the ROC curve in Figure 13, is also reported for the CoV-NonCoV-15k dataset because of its imbalanced nature.

ROC and PR analysis evidence that the proposed Channel Boosting based CB-STM-RENet at different cutoffs shows significant detection performance. Figure 12 and Figure 13 show that our proposed technique with Channel Boosting achieved an AUC-ROC of 0.99 on both the datasets (CoV-Healthy-6k and CoV-NonCoV-10k) and 0.98 AUC-PR for CoV-NonCoV15k. The high value of AUC demonstrates that the Channel Boosting based CB-STM-RENet upholds high sensitivity and precision and performs well as a whole for COVID19 patients’ screening.

## 5. Conclusions

COVID-19 is a contagious viral infectious disease that has affected a large spectrum of the population globally. Its high transmissibility and pathogenic nature make the early detection of COVID-19 indispensable for infection control and stoppage. Radiographic images exhibit COVID-19 specific patterns such as Ground Glass Opacity, Consolidation, Reticulation, and blurring of lung markings compared to healthy individuals. This work proposes STM-RENet, which incorporates the idea of classical image processing in convolutional operators to explore region homogeneity, boundary patterns, and textural variations, whereas the idea of Channel Boosting is incorporated to aggregate multi-level radiographic patterns. The empirical evaluation of the proposed technique suggests that it performs significantly well on a stringent dataset with an F-score of 0.95 and accuracy of 96.53%, and surpasses the performance of existing techniques with a 7% improvement. This suggests that the proposed technique can effectively discriminate COVID-19 chest infections from Healthy individuals and other types of chest infections. In the future, the proposed CB-STM-RENet will be exploited for the multi-class segregation of COVID-like MERS and SARS-CoV, etc., and new variants of COVID-19, such as Omicron.

## Figures and Tables

**Table 1:** Performance analysis of the proposed techniques on test dataset (CoV-Healthy-6k, CoV-NonCoV-10k and CoV-NonCoV-15k). S.E at 95% CI is reported for sensitivity.

		**CoV-Healthy-6k**				
**Techniques**	**Accuracy %**	**MCC**	**F-Score**	**Sensitivity ± S.E**	**Precision**	**Specificity**
**CB-STM-RENet**	**98.53**	**0.97**	**0.98**	**0.99 ± 0.02**	**0.98**	**0.98**
**STM-RENet**	97.98	0.96	0.98	0.97 ± 0.04	0.99	0.99
		**CoV-NonCoV-10k**				
**CB-STM-RENet**	**97.48**	**0.95**	**0.98**	**0.99 ± 0.02**	**0.98**	0.96
**STM-RENet**	91.82	0.84	0.92	0.97 ± 0.04	0.88	0.86
		**CoV-NonCoV-15k**				
**CB-STM-RENet**	**96.53**	**0.93**	**0.95**	**0.97 ± 0.04**	**0.93**	**0.96**
**STM-RENet**	92.04	0.85	0.90	0.96 ± 0.05	0.84	0.90

**Table 2:** Ablation study for the proposed deep CNN models on CoV-Healthy-6k dataset.

Networks	Accuracy %	MCC	F-Score	Sensitivity ± S.E	Precision	Specificity	TP	FP	FN	TN
**CB-STM-RENet**	**98.53**	**0.97**	**0.98**	**0.99 ± 0.02**	**0.98**	**0.98**	**638**	**12**	**7**	**633**
**CB-STM-RENet (Auxiliary learner 2)**	98.22	0.96	0.98	0.98 ± 0.03	0.99	0.99	**631**	9	**14**	636
**CB-STM-RENet (Auxiliary learner 1)**	98.14	0.96	0.98	0.98 ± 0.03	0.98	0.98	**634**	13	**11**	632
**STM-RENet**	97.98	0.96	0.98	0.97 ± 0.04	0.99	0.99	**625**	6	**20**	639

**Table 3:** Performance analysis of the best performing existing CNN techniques on the test set of CoV-Healthy-6k, CoV-NonCoV-10k and CoV-NonCoV-15k. S.E is reported for sensitivity.

		**CoV-Healthy-6k**				
**Techniques**	**Accuracy %**	**MCC**	**F-Score**	**Sensitivity ± S.E**	**Precision**	**Specificity**
**Resnet18**	96.59	0.93	0.96	0.98 ± 0.03	0.95	0.95
**VGG-16**	95.74	0.91	0.95	0.96 ± 0.05	0.95	0.95
		**CoV-NonCoV-10k**				
**ResNet-18**	**90.36**	**0.81**	**0.90**	0.90 ± 0.12	**0.90**	**0.91**
**VGG-16**	86.32	0.73	0.86	0.86 ± 0.16	0.86	0.87
		**CoV-NonCoV-15k**				
**ResNet-18**	**91.20**	**0.84**	**0.88**	0.95 ± 0.06	**0.82**	**0.89**
**VGG-16**	88.26	0.77	0.84	0.89 ± 0.13	0.80	0.88

**Table 4:** Performance of existing CNN techniques on the test set of CoV-Healthy-6k. S.E is reported for sensitivity.

Techniques	Trained from Scratch and TL-Based Fine-Tuned CNNs					
	Accuracy %	MCC	F-Score	Sensitivity ± S.E	Precision	Specificity
**ShuffleNet**	84.88	0.75	0.86	0.95 ± 0.06	0.79	0.75
**TL_ShuffleNet**	96.59	0.93	0.96	0.96 ± 0.05	0.97	0.97
**Inceptionv3**	93.80	0.86	0.93	0.95 ± 0.06	0.92	0.92
**TL_Inceptionv3**	96.51	0.93	0.96	0.97 ± 0.04	0.96	0.96
**Alexnet**	94.50	0.89	0.94	0.92 ± 0.09	0.97	0.97
**TL_Alexnet**	95.74	0.91	0.95	0.96 ± 0.05	0.95	0.95
**DenseNet201**	95.50	0.91	0.95	0.95 ± 0.06	0.96	0.96
**TL_DenseNet201**	96.51	0.93	0.96	0.96 ± 0.05	0.97	0.97
**Xception**	95.74	0.91	0.95	0.94 ± 0.07	0.97	0.97
**TL_Xception**	96.43	0.93	0.96	0.96 ± 0.05	0.97	0.97
**TL_ VGG_16**	97.05	0.94	0.97	0.97 ± 0.04	0.97	0.97
**Resnet50**	96.28	0.92	0.96	0.95 ± 0.06	0.98	0.98
**TL_Resnet50**	97.05	0.94	0.97	0.97 ± 0.04	0.97	0.98
**TL_Resnet18**	97.13	0.94	0.97	0.97 ± 0.04	0.98	0.98

**Table 5:** Performance analysis of the reported CNNs on the test set of CoV-NonCoV-15k. S.E at 95% CI is reported for sensitivity.

CoV-NonCoV-15k						
Techniques	Accuracy %	MCC	F-Score	Sensitivity ± S.E	Precision	Specificity
**SqueezeNet**	83.76	0.62	0.75	0.69 ± 0.33	0.81	0.92
**ResNet-50**	86.84	0.69	0.80	0.74 ± 0.28	0.86	0.94
**DenseNet-201**	89.58	0.77	0.85	0.84 ± 0.17	0.86	0.93

## Data Availability

We have shared the related code and materials at https://github.com/PRLAB21/COVID-19-Detection-System-using-Chest-X-ray-Images accessed on 12 December 2021.

## Abbreviations

Coronavirus (CoV), Polymerase Chain Reaction (PCR), ground-glass opacities (GGO), Deep Learning (DL), Transfer Learning (TL), Channel Boosted (CB), split-transform-merge (STM).
